# 2-(4-Bromo­phen­yl)-1-(phenyl­sulfin­yl)naphtho[2,1-*b*]furan

**DOI:** 10.1107/S1600536809029250

**Published:** 2009-07-29

**Authors:** Hong Dae Choi, Pil Ja Seo, Byeng Wha Son, Uk Lee

**Affiliations:** a Department of Chemistry, Dongeui University, San 24 Kaya-dong Busanjin-gu, Busan 614-714, Republic of Korea; b Department of Chemistry, Pukyong National University, 599-1 Daeyeon 3-dong, Nam-gu, Busan 608-737, Republic of Korea

## Abstract

In the title compound, C_24_H_15_BrO_2_S, the sulfinyl O atom and the phenyl group of the phenyl­sulfinyl substituent lie on opposite sides of the plane through the naphthofuran fragment. The phenyl ring is nearly perpendicular to the plane of the tricyclic naphthofuran system [81.77 (6)°] and is tilted slightly towards it. The 4-bromo­phenyl ring is rotated out of the naphthofuran plane by a dihedral angle of 31.12 (4)°. In the crystal structure, non-classical inter­molecular C—H⋯O and C—H⋯Br hydrogen bonds are observed. The crystal structure also exhibits aromatic π–π inter­actions between the furan ring and the central benzene ring of the adjacent naphthofuran system [centroid–centroid distance = 3.768 (3) Å]. In addition, inter­molecular C—Br⋯π inter­actions [3.866 (2) Å] between the Br atom and the phenyl ring of the phenyl­sulfinyl substituent are present.

## Related literature

For the crystal structures of similar 2-phenyl-1-(phenyl­sulfin­yl)-naphtho[2,1-*b*]furan derivatives, see: Choi *et al.* (2009*a* ▶,*b* ▶). For the biological and pharmacological activity of naphthofuran compounds, see: Goel & Dixit (2004 ▶); Hagiwara *et al.* (1999 ▶); Piloto *et al.* (2005 ▶).
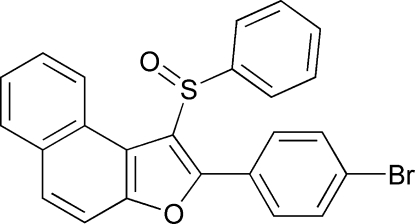


## Experimental

### 

#### Crystal data

C_24_H_15_BrO_2_S
*M* *_r_* = 447.33
Triclinic, 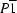

*a* = 9.2412 (5) Å
*b* = 10.3266 (6) Å
*c* = 10.7606 (6) Å
α = 71.424 (1)°
β = 77.933 (1)°
γ = 79.287 (1)°
*V* = 943.96 (9) Å^3^
*Z* = 2
Mo *K*α radiation
μ = 2.31 mm^−1^
*T* = 273 K
0.50 × 0.20 × 0.15 mm

#### Data collection

Bruker SMART CCD diffractometer
Absorption correction: multi-scan (*SADABS*; Sheldrick, 2000 ▶) *T*_min_ = 0.392, *T*_max_ = 0.724
8200 measured reflections
4057 independent reflections
3482 reflections with *I* > 2σ(*I*)
*R*_int_ = 0.014

#### Refinement

*R*[*F* ^2^ > 2σ(*F* ^2^)] = 0.026
*wR*(*F* ^2^) = 0.068
*S* = 1.06
4057 reflections
253 parameters
H-atom parameters constrained
Δρ_max_ = 0.29 e Å^−3^
Δρ_min_ = −0.33 e Å^−3^

### 

Data collection: *SMART* (Bruker, 2001 ▶); cell refinement: *SAINT* (Bruker, 2001 ▶); data reduction: *SAINT*; program(s) used to solve structure: *SHELXS97* (Sheldrick, 2008 ▶); program(s) used to refine structure: *SHELXL97* (Sheldrick, 2008 ▶); molecular graphics: *ORTEP-3* (Farrugia, 1997 ▶) and *DIAMOND* (Brandenburg, 1998 ▶); software used to prepare material for publication: *SHELXL97*.

## Supplementary Material

Crystal structure: contains datablocks global, I. DOI: 10.1107/S1600536809029250/im2130sup1.cif

Structure factors: contains datablocks I. DOI: 10.1107/S1600536809029250/im2130Isup2.hkl

Additional supplementary materials:  crystallographic information; 3D view; checkCIF report

## Figures and Tables

**Table 1:** Hydrogen-bond geometry (Å, °)

*D*—H⋯*A*	*D*—H	H⋯*A*	*D*⋯*A*	*D*—H⋯*A*
C7—H7⋯O2^i^	0.93	2.57	3.482 (2)	167
C20—H20⋯Br^ii^	0.93	2.98	3.760 (2)	143

Symmetry codes: (i) 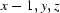; (ii) 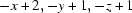.

**Fig. 1.**
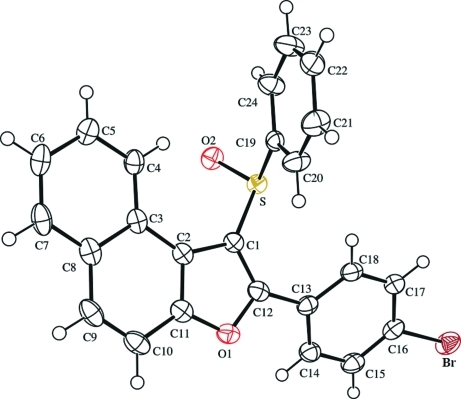
Molecular structure of the title compound with the atom numbering scheme. Displacement ellipsoids are drawn at the 30% probability level. H atoms are presented as a small spheres of arbitrary radius.

**Fig. 2.**
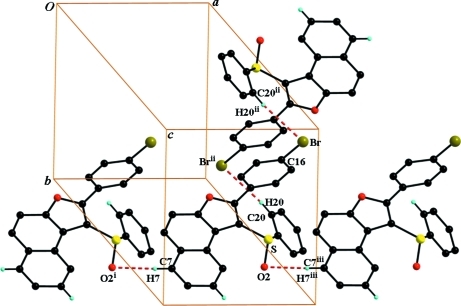
The C–H···O and C–H···Br interactions (dotted lines) in the title compound. [Symmetry code: (i) x - 1, y, z; (ii) - x + 2, - y + 1, - z + 1; (iii) x + 1, y, z.]

**Fig. 3.**
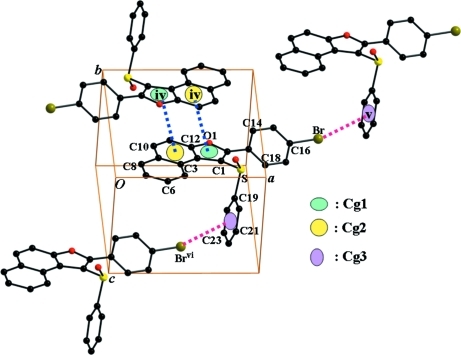
The π–π and C–Br···π interactions (dotted lines) in the title compound. Cg denotes the ring centroids. [Symmetry code: (iv) - x + 1, - y + 2, - z + 1; (v) x + 1, y, z - 1; (vi) x-1, y, z + 1.]

## supplementary crystallographic information

### Comment

Molecules containing naphthofuran moieties have attracted considerable interest in view of their biological and pharmacological activities (Goel & Dixit, 2004; Hagiwara *et al.*, 1999; Piloto *et al.*, 2005). This work is related to our communications on the synthesis and structures of 2-phenyl-1-(phenylsulfinyl)naphtho[2,1-b]furan analogues, as 2-phenyl-1-(phenylsulfinyl)naphtho[2,1-b]furan (Choi *et al.*, 2009a) and 7-bromo-2-phenyl-1-(phenylsulfinyl)naphtho[2,1-b]furan (Choi *et al.*, 2009b). Here we report the crystal structure of the title compound (I) (Fig. 1).

The naphthofuran unit is essentially planar, with a mean deviation of 0.020 (2) Å from the least-squares plane defined by the thirteen constituent atoms. The dihedral angle in (I) formed by the plane of the naphthofuran system and the plane of the 4-bromophenyl ring measures to 31.12 (4) Å. The respective dihedral angle with the phenyl ring (C19-C24) shows a value of 81.77 (6) Å with respect to the naphthofuran plane. The crystal packing (Fig. 2) is realized by non-classical intermolecular C–H···O and C–H···Br hydrogen bonds (Table 1). In the crystal structure (Fig. 3) additionally aromatic π–π interactions between the furan ring and the central benzene ring of adjacent molecules are observed. The Cg1···Cg2^iv^ distance is 3.768 (3) Å (Cg1 and Cg2 are the centroides of the C1/C2/C11/O1/C12 furan and the C2/C3/C8/C9/C10/C11 benzene rings, respectively). The molecular packing (Fig. 3) also exhibits intermolecular C–Br···π interactions between the Br atom and the phenyl ring of the phenylsulfinyl substituent, with a C16–Br···Cg3^v^ (3.866 (2) Å ; Cg3 is the centroid of C19-C24 benzene ring).

### Experimental

3-Chloroperoxybenzoic acid (77%, 157 mg, 0.7 mmol) was added in small portions to a stirred solution of 2-(4-bromophenyl)-1-(phenylsulfanyl)naphtho[2,1-b]furan (313 mg, 0.7 mmol) in dichloromethane (40 mL) at 273 K. After being stirred at room temperature for 4h, the mixture was washed with saturated sodium bicarbonate solution and the organic layer was separated, dried over magnesium sulfate, filtered and concentrated in vacuum. The residue was purified by column chromatography (hexane-ethyl acetate, 2:1 v/v) to afford the title compound as a colorless solid (yield 78%, m.p. 447-448 K; *R*_f_ = 0.61 (hexane-ethyl acetate, 2:1 v/v)). Single crystals suitable for X-ray diffraction were prepared by slow evaporation of a solution of the title compound in benzene at room temperature.

### Refinement

All H atoms were positioned geometrically and refined using a riding model, with C–H = 0.93 Å and with *U*_iso_(H) = 1.2*U*_eq_(C) for aromatic H atoms.

### Crystal data

**Table:**

C_24_H_15_BrO_2_S	*Z* = 2
*M**_r_* = 447.33	*F*(000) = 452
Triclinic, *P*1	*D*_x_ = 1.574 Mg m^−^^3^
Hall symbol: -P 1	Mo *K*α radiation, λ = 0.71073 Å
*a* = 9.2412 (5) Å	Cell parameters from 4680 reflections
*b* = 10.3266 (6) Å	θ = 2.3–27.5°
*c* = 10.7606 (6) Å	µ = 2.31 mm^−^^1^
α = 71.424 (1)°	*T* = 273 K
β = 77.933 (1)°	Block, colorless
γ = 79.287 (1)°	0.50 × 0.20 × 0.15 mm
*V* = 943.96 (9) Å^3^	

### Data collection

**Table:**

Bruker SMART CCD diffractometer	4057 independent reflections
Radiation source: fine-focus sealed tube	3482 reflections with *I* > 2σ(*I*)
graphite	*R*_int_ = 0.014
Detector resolution: 10.0 pixels mm^-1^	θ_max_ = 27.0°, θ_min_ = 2.0°
φ and ω scans	*h* = −11→11
Absorption correction: multi-scan (*SADABS*; Sheldrick, 2000)	*k* = −13→12
*T*_min_ = 0.392, *T*_max_ = 0.724	*l* = −13→13
8200 measured reflections	

### Refinement

**Table:**

Refinement on *F*^2^	Primary atom site location: structure-invariant direct methods
Least-squares matrix: full	Secondary atom site location: difference Fourier map
*R*[*F*^2^ > 2σ(*F*^2^)] = 0.026	Hydrogen site location: difference Fourier map
*wR*(*F*^2^) = 0.068	H-atom parameters constrained
*S* = 1.06	*w* = 1/[σ^2^(*F*_o_^2^) + (0.0322*P*)^2^ + 0.4094*P*] where *P* = (*F*_o_^2^ + 2*F*_c_^2^)/3
4057 reflections	(Δ/σ)_max_ = 0.001
253 parameters	Δρ_max_ = 0.29 e Å^−^^3^
0 restraints	Δρ_min_ = −0.33 e Å^−^^3^

### Special details

**Table:**

Geometry. All esds (except the esd in the dihedral angle between two l.s. planes) are estimated using the full covariance matrix. The cell esds are taken into account individually in the estimation of esds in distances, angles and torsion angles; correlations between esds in cell parameters are only used when they are defined by crystal symmetry. An approximate (isotropic) treatment of cell esds is used for estimating esds involving l.s. planes.
Refinement. Refinement of F^2^ against ALL reflections. The weighted R-factor wR and goodness of fit S are based on F^2^, conventional R-factors R are based on F, with F set to zero for negative F^2^. The threshold expression of F^2^ > 2sigma(F^2^) is used only for calculating R-factors(gt) etc. and is not relevant to the choice of reflections for refinement. R-factors based on F^2^ are statistically about twice as large as those based on F, and R- factors based on ALL data will be even larger.

### Fractional atomic coordinates and isotropic or equivalent isotropic displacement parameters (Å^2^)

**Table:**

	*x*	*y*	*z*	*U*_iso_*/*U*_eq_	
Br	1.42799 (2)	0.59150 (2)	0.28002 (2)	0.04232 (8)	
S	0.86347 (5)	0.80600 (5)	0.75366 (4)	0.02428 (10)	
O1	0.67887 (14)	0.76607 (13)	0.47329 (12)	0.0288 (3)	
O2	0.81883 (15)	0.94123 (13)	0.78284 (13)	0.0311 (3)	
C1	0.73992 (19)	0.78681 (18)	0.65760 (17)	0.0242 (4)	
C2	0.57973 (19)	0.82284 (18)	0.66420 (17)	0.0246 (4)	
C3	0.45921 (19)	0.86444 (18)	0.75651 (18)	0.0256 (4)	
C4	0.4754 (2)	0.88062 (19)	0.87792 (19)	0.0290 (4)	
H4	0.5700	0.8656	0.9009	0.035*	
C5	0.3534 (2)	0.9183 (2)	0.9630 (2)	0.0328 (4)	
H5	0.3659	0.9284	1.0428	0.039*	
C6	0.2097 (2)	0.9413 (2)	0.9292 (2)	0.0350 (4)	
H6	0.1275	0.9660	0.9873	0.042*	
C7	0.1900 (2)	0.92773 (19)	0.8119 (2)	0.0342 (4)	
H7	0.0943	0.9434	0.7912	0.041*	
C8	0.3132 (2)	0.89007 (18)	0.7210 (2)	0.0293 (4)	
C9	0.2929 (2)	0.8767 (2)	0.5981 (2)	0.0334 (4)	
H9	0.1972	0.8955	0.5768	0.040*	
C10	0.4087 (2)	0.83736 (19)	0.5108 (2)	0.0330 (4)	
H10	0.3946	0.8296	0.4306	0.040*	
C11	0.5507 (2)	0.80912 (18)	0.54840 (18)	0.0277 (4)	
C12	0.7932 (2)	0.75313 (18)	0.54145 (18)	0.0262 (4)	
C13	0.9424 (2)	0.70872 (18)	0.47959 (17)	0.0259 (4)	
C14	0.9808 (2)	0.74611 (19)	0.34116 (18)	0.0306 (4)	
H14	0.9093	0.7958	0.2886	0.037*	
C15	1.1239 (2)	0.7101 (2)	0.28158 (19)	0.0333 (4)	
H15	1.1488	0.7356	0.1895	0.040*	
C16	1.2295 (2)	0.6357 (2)	0.36050 (19)	0.0298 (4)	
C17	1.1936 (2)	0.5935 (2)	0.49761 (19)	0.0306 (4)	
H17	1.2651	0.5419	0.5494	0.037*	
C18	1.0501 (2)	0.62905 (19)	0.55646 (18)	0.0287 (4)	
H18	1.0248	0.5996	0.6484	0.034*	
C19	0.81395 (18)	0.67454 (18)	0.90586 (17)	0.0234 (3)	
C24	0.8333 (2)	0.6950 (2)	1.0216 (2)	0.0345 (4)	
H24	0.8639	0.7766	1.0198	0.041*	
C20	0.7690 (2)	0.5535 (2)	0.90810 (19)	0.0350 (4)	
H20	0.7560	0.5403	0.8298	0.042*	
C21	0.7434 (3)	0.4515 (2)	1.0279 (2)	0.0379 (5)	
H21	0.7124	0.3700	1.0300	0.045*	
C22	0.7637 (2)	0.4706 (2)	1.1437 (2)	0.0342 (4)	
H22	0.7485	0.4015	1.2236	0.041*	
C23	0.8065 (3)	0.5924 (2)	1.1409 (2)	0.0408 (5)	
H23	0.8176	0.6061	1.2196	0.049*	

### Atomic displacement parameters (Å^2^)

**Table:**

	*U*^11^	*U*^22^	*U*^33^	*U*^12^	*U*^13^	*U*^23^
Br	0.03273 (12)	0.05681 (15)	0.04193 (13)	−0.00596 (9)	0.00269 (9)	−0.02602 (11)
S	0.0218 (2)	0.0270 (2)	0.0262 (2)	−0.00630 (17)	−0.00274 (16)	−0.00963 (17)
O1	0.0327 (7)	0.0293 (7)	0.0271 (6)	−0.0064 (5)	−0.0082 (5)	−0.0083 (5)
O2	0.0335 (7)	0.0263 (7)	0.0368 (7)	−0.0081 (5)	−0.0050 (6)	−0.0117 (6)
C1	0.0242 (9)	0.0234 (9)	0.0258 (9)	−0.0049 (7)	−0.0049 (7)	−0.0065 (7)
C2	0.0244 (9)	0.0211 (8)	0.0283 (9)	−0.0059 (7)	−0.0063 (7)	−0.0043 (7)
C3	0.0225 (8)	0.0191 (8)	0.0342 (10)	−0.0052 (7)	−0.0054 (7)	−0.0044 (7)
C4	0.0222 (9)	0.0275 (9)	0.0378 (10)	−0.0026 (7)	−0.0042 (8)	−0.0109 (8)
C5	0.0281 (9)	0.0309 (10)	0.0409 (11)	−0.0041 (8)	−0.0012 (8)	−0.0150 (8)
C6	0.0225 (9)	0.0276 (10)	0.0526 (12)	−0.0018 (7)	0.0015 (8)	−0.0139 (9)
C7	0.0215 (9)	0.0225 (9)	0.0569 (13)	−0.0032 (7)	−0.0091 (8)	−0.0071 (9)
C8	0.0259 (9)	0.0189 (9)	0.0420 (11)	−0.0062 (7)	−0.0083 (8)	−0.0034 (8)
C9	0.0288 (10)	0.0266 (10)	0.0453 (12)	−0.0078 (8)	−0.0166 (9)	−0.0018 (8)
C10	0.0368 (11)	0.0280 (10)	0.0375 (11)	−0.0101 (8)	−0.0167 (9)	−0.0039 (8)
C11	0.0305 (9)	0.0226 (9)	0.0301 (9)	−0.0071 (7)	−0.0073 (7)	−0.0044 (7)
C12	0.0313 (9)	0.0216 (9)	0.0254 (9)	−0.0062 (7)	−0.0073 (7)	−0.0031 (7)
C13	0.0325 (9)	0.0214 (9)	0.0252 (9)	−0.0063 (7)	−0.0029 (7)	−0.0080 (7)
C14	0.0394 (10)	0.0257 (9)	0.0261 (9)	−0.0035 (8)	−0.0073 (8)	−0.0056 (7)
C15	0.0432 (11)	0.0319 (10)	0.0232 (9)	−0.0078 (8)	−0.0005 (8)	−0.0072 (8)
C16	0.0285 (9)	0.0320 (10)	0.0327 (10)	−0.0077 (8)	0.0009 (8)	−0.0163 (8)
C17	0.0340 (10)	0.0297 (10)	0.0305 (10)	−0.0007 (8)	−0.0083 (8)	−0.0120 (8)
C18	0.0368 (10)	0.0275 (9)	0.0227 (9)	−0.0050 (8)	−0.0042 (8)	−0.0085 (7)
C19	0.0194 (8)	0.0249 (9)	0.0265 (9)	−0.0010 (6)	−0.0045 (7)	−0.0087 (7)
C24	0.0450 (11)	0.0311 (10)	0.0329 (10)	−0.0109 (9)	−0.0146 (9)	−0.0085 (8)
C20	0.0500 (12)	0.0320 (10)	0.0270 (10)	−0.0104 (9)	−0.0055 (9)	−0.0117 (8)
C21	0.0525 (13)	0.0269 (10)	0.0360 (11)	−0.0115 (9)	−0.0038 (9)	−0.0100 (8)
C22	0.0388 (11)	0.0305 (10)	0.0289 (10)	−0.0020 (8)	−0.0056 (8)	−0.0038 (8)
C23	0.0583 (14)	0.0400 (12)	0.0282 (10)	−0.0096 (10)	−0.0168 (10)	−0.0075 (9)

### Geometric parameters (Å, °)

**Table:**

Br—C16	1.8973 (19)	C10—H10	0.9300
S—O2	1.4933 (13)	C12—C13	1.460 (3)
S—C1	1.7662 (18)	C13—C14	1.399 (3)
S—C19	1.7987 (18)	C13—C18	1.400 (3)
O1—C12	1.371 (2)	C14—C15	1.382 (3)
O1—C11	1.373 (2)	C14—H14	0.9300
C1—C12	1.372 (2)	C15—C16	1.384 (3)
C1—C2	1.450 (2)	C15—H15	0.9300
C2—C11	1.382 (3)	C16—C17	1.384 (3)
C2—C3	1.429 (2)	C17—C18	1.382 (3)
C3—C4	1.410 (3)	C17—H17	0.9300
C3—C8	1.434 (3)	C18—H18	0.9300
C4—C5	1.375 (3)	C19—C24	1.380 (3)
C4—H4	0.9300	C19—C20	1.381 (3)
C5—C6	1.408 (3)	C24—C23	1.390 (3)
C5—H5	0.9300	C24—H24	0.9300
C6—C7	1.365 (3)	C20—C21	1.388 (3)
C6—H6	0.9300	C20—H20	0.9300
C7—C8	1.419 (3)	C21—C22	1.376 (3)
C7—H7	0.9300	C21—H21	0.9300
C8—C9	1.426 (3)	C22—C23	1.376 (3)
C9—C10	1.361 (3)	C22—H22	0.9300
C9—H9	0.9300	C23—H23	0.9300
C10—C11	1.405 (3)		
			
O2—S—C1	109.42 (8)	O1—C12—C13	116.43 (15)
O2—S—C19	107.01 (8)	C1—C12—C13	133.04 (17)
C1—S—C19	99.88 (8)	C14—C13—C18	118.53 (17)
C12—O1—C11	106.49 (14)	C14—C13—C12	120.29 (17)
C12—C1—C2	106.91 (15)	C18—C13—C12	121.17 (16)
C12—C1—S	120.79 (14)	C15—C14—C13	120.79 (18)
C2—C1—S	131.42 (14)	C15—C14—H14	119.6
C11—C2—C3	119.50 (16)	C13—C14—H14	119.6
C11—C2—C1	104.65 (16)	C14—C15—C16	119.25 (17)
C3—C2—C1	135.85 (17)	C14—C15—H15	120.4
C4—C3—C2	124.40 (16)	C16—C15—H15	120.4
C4—C3—C8	119.09 (17)	C17—C16—C15	121.29 (18)
C2—C3—C8	116.51 (17)	C17—C16—Br	119.08 (15)
C5—C4—C3	121.00 (17)	C15—C16—Br	119.63 (14)
C5—C4—H4	119.5	C18—C17—C16	119.12 (18)
C3—C4—H4	119.5	C18—C17—H17	120.4
C4—C5—C6	119.96 (19)	C16—C17—H17	120.4
C4—C5—H5	120.0	C17—C18—C13	120.93 (17)
C6—C5—H5	120.0	C17—C18—H18	119.5
C7—C6—C5	120.60 (18)	C13—C18—H18	119.5
C7—C6—H6	119.7	C24—C19—C20	120.56 (17)
C5—C6—H6	119.7	C24—C19—S	116.87 (14)
C6—C7—C8	121.14 (18)	C20—C19—S	122.40 (14)
C6—C7—H7	119.4	C19—C24—C23	119.21 (18)
C8—C7—H7	119.4	C19—C24—H24	120.4
C7—C8—C9	121.21 (18)	C23—C24—H24	120.4
C7—C8—C3	118.20 (18)	C19—C20—C21	119.64 (18)
C9—C8—C3	120.59 (18)	C19—C20—H20	120.2
C10—C9—C8	122.32 (18)	C21—C20—H20	120.2
C10—C9—H9	118.8	C22—C21—C20	120.15 (19)
C8—C9—H9	118.8	C22—C21—H21	119.9
C9—C10—C11	116.50 (18)	C20—C21—H21	119.9
C9—C10—H10	121.7	C23—C22—C21	119.87 (19)
C11—C10—H10	121.7	C23—C22—H22	120.1
O1—C11—C2	111.42 (16)	C21—C22—H22	120.1
O1—C11—C10	124.05 (17)	C22—C23—C24	120.55 (19)
C2—C11—C10	124.53 (18)	C22—C23—H23	119.7
O1—C12—C1	110.53 (16)	C24—C23—H23	119.7
			
O2—S—C1—C12	128.31 (15)	C9—C10—C11—C2	−2.1 (3)
C19—S—C1—C12	−119.58 (15)	C11—O1—C12—C1	0.08 (19)
O2—S—C1—C2	−39.51 (19)	C11—O1—C12—C13	−179.65 (15)
C19—S—C1—C2	72.60 (18)	C2—C1—C12—O1	0.4 (2)
C12—C1—C2—C11	−0.79 (19)	S—C1—C12—O1	−170.03 (12)
S—C1—C2—C11	168.29 (14)	C2—C1—C12—C13	−179.88 (18)
C12—C1—C2—C3	178.88 (19)	S—C1—C12—C13	9.6 (3)
S—C1—C2—C3	−12.0 (3)	O1—C12—C13—C14	32.4 (2)
C11—C2—C3—C4	179.44 (17)	C1—C12—C13—C14	−147.3 (2)
C1—C2—C3—C4	−0.2 (3)	O1—C12—C13—C18	−148.06 (17)
C11—C2—C3—C8	−0.2 (2)	C1—C12—C13—C18	32.3 (3)
C1—C2—C3—C8	−179.86 (19)	C18—C13—C14—C15	−2.7 (3)
C2—C3—C4—C5	−178.61 (17)	C12—C13—C14—C15	176.95 (17)
C8—C3—C4—C5	1.0 (3)	C13—C14—C15—C16	0.3 (3)
C3—C4—C5—C6	−0.1 (3)	C14—C15—C16—C17	1.8 (3)
C4—C5—C6—C7	−0.5 (3)	C14—C15—C16—Br	−177.73 (15)
C5—C6—C7—C8	0.0 (3)	C15—C16—C17—C18	−1.4 (3)
C6—C7—C8—C9	−179.59 (18)	Br—C16—C17—C18	178.17 (14)
C6—C7—C8—C3	1.0 (3)	C16—C17—C18—C13	−1.1 (3)
C4—C3—C8—C7	−1.5 (3)	C14—C13—C18—C17	3.1 (3)
C2—C3—C8—C7	178.21 (16)	C12—C13—C18—C17	−176.49 (17)
C4—C3—C8—C9	179.06 (17)	O2—S—C19—C24	−37.46 (17)
C2—C3—C8—C9	−1.2 (3)	C1—S—C19—C24	−151.42 (15)
C7—C8—C9—C10	−178.31 (18)	O2—S—C19—C20	147.24 (16)
C3—C8—C9—C10	1.1 (3)	C1—S—C19—C20	33.28 (18)
C8—C9—C10—C11	0.5 (3)	C20—C19—C24—C23	−0.2 (3)
C12—O1—C11—C2	−0.62 (19)	S—C19—C24—C23	−175.62 (17)
C12—O1—C11—C10	178.53 (17)	C24—C19—C20—C21	−0.1 (3)
C3—C2—C11—O1	−178.87 (15)	S—C19—C20—C21	175.04 (16)
C1—C2—C11—O1	0.9 (2)	C19—C20—C21—C22	−0.5 (3)
C3—C2—C11—C10	2.0 (3)	C20—C21—C22—C23	1.4 (3)
C1—C2—C11—C10	−178.27 (17)	C21—C22—C23—C24	−1.7 (3)
C9—C10—C11—O1	178.85 (16)	C19—C24—C23—C22	1.1 (3)

### Hydrogen-bond geometry (Å, °)

**Table:**

*D*—H···*A*	*D*—H	H···*A*	*D*···*A*	*D*—H···*A*
C7—H7···O2^i^	0.93	2.57	3.482 (2)	167
C20—H20···Br^ii^	0.93	2.98	3.760 (2)	143

Symmetry codes: (i) *x*−1, *y*, *z*; (ii) −*x*+2, −*y*+1, −*z*+1.
